# Draft Genome Sequence of Bacillus thuringiensis Strain UNMSM10RA, Isolated from Potato Crop Soil in Peru

**DOI:** 10.1128/MRA.01189-19

**Published:** 2020-01-09

**Authors:** Yolanda Bedsabé Delgado-Silva, David Tarazona, Fernando Serna, Eduardo Juscamayta, Julio César Chávez-Galarza, Evelyn Roxana Farfán-Vignolo, Gabriel Delgado, Abad Flores, Gabriela Solano, Dina L. Gutierrez

**Affiliations:** a Dirección de Recursos Genéticos y Biotecnología, Instituto Nacional de Innovación Agraria (INIA), La Molina, Lima, Perú; b Dirección de Desarrollo Tecnológico Agrario, Instituto Nacional de Innovación Agraria (INIA), La Molina, Lima, Perú; c Laboratorio de Microbiología Ambiental y Biotecnología, Facultad de Ciencias Biológicas, Universidad Nacional Mayor de San Marcos, Ciudad Universitaria, Lima, Perú; d Escuela Académico Profesional de Agronomía, Universidad Nacional de Cañete, Urb. Santa Rosa de Hualcará, San Vicente de Cañete, Lima, Perú; Indiana University, Bloomington

## Abstract

The 5.5-Mb genome sequence of Bacillus thuringiensis strain UNMSM10RA, isolated from potato crop soil, is reported in this study. The strain UNMSM10RA contains 5,347 protein-coding sequences, 105 tRNA genes, 15 rRNA genes, and 5 noncoding RNA (ncRNA) genes, with an average G+C content of 35.1%.

## ANNOUNCEMENT

Bacillus thuringiensis is an aerobic, ubiquitous, rod-shaped, Gram-positive, spore-forming bacterium that has been isolated from a variety of environments. This bacterium is well known for producing parasporal crystalline inclusions containing Cry and Cyt proteins (also known as δ-endotoxins) (1). Currently, the sequencing of Bacillus thuringiensis strain genomes has increased and facilitated the search of not only new molecules with insecticidal activity but also genes conferring the ability for heavy metal biosorption, resistance, or tolerance (2).

We isolated Bacillus thuringiensis strains from potato crop soils in Huaral, Lima, Peru. Serial dilutions (10^−3^) were done for each sample in a peptone salt solution and incubated at 70°C for 15 min, followed by thermal shock. Then, the samples were incubated at 70°C for 20 min, followed by incubation at 30°C for 18 h. Positives samples were cultured overnight at 30°C in Luria-Bertani (LB) medium under aerobic conditions. One of the colonies recovered, named UNMSM10RA, was chosen for sequencing.

For UNMSM10RA genome sequencing, total genomic DNA was extracted from overnight cultures using the High Pure PCR template preparation kit (Roche, USA) according to the manufacturer’s instructions. Genomic DNA was used for library preparation with the Nextera XT DNA library preparation kit. The library showed a size range of ∼250 to 1,000 bp. Whole-genome sequencing was performed on an Illumina MiSeq system (San Diego, CA) in a paired-end mode with a read length of 150 bp. A total of 969,034 reads were generated. Quality control of these reads was performed with the FastQC tool (http://www.bioinformatics.babraham.ac.uk/projects/fastqc/). *De novo* genome assembly was done using Velvet v.1.2.10 (3) and SPAdes v.3.12.1 (4) and merged for the best assembly with genomic assemblies merger for next-generation sequencing (GAM-NGS) through the PATRIC server on 22 January 2018 (5). Genome annotation was carried out using the Rapid Annotations using Subsystems Technology (RAST) platform (6), NCBI Prokaryotic Genome Automatic Annotation Pipeline (PGAAP), and Clusters of Orthologous Groups (7). tRNA and rRNA were predicted by tRNAscan-SE (8) and RNAmmer (9), respectively. *Bacillus* species confirmation was performed by the comparison of genome-derived 16S rRNA sequences and establishing their phylogenetic relationships using MEGA X software (10). All programs were set up with default parameters.

**FIG 1:**
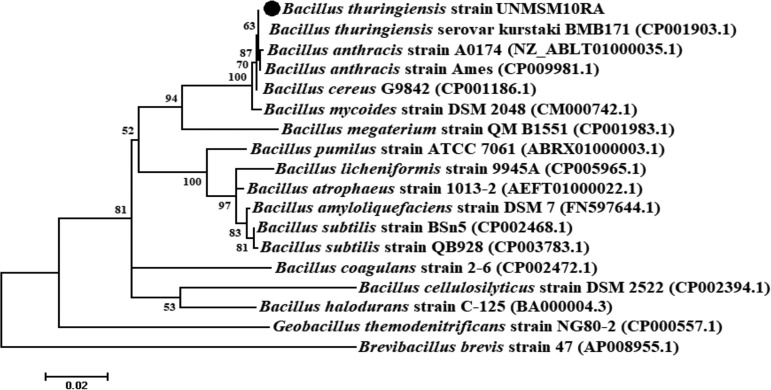
Phylogenetic relationships based on 16S rRNA sequences highlighting the position of Bacillus thuringiensis strain UNMSM10RA and other species of *Bacillus* and related genera. GenBank accession numbers are presented in parentheses. This topology was inferred from a multiple alignment using ClustalW 2.1 and the maximum likelihood method within MEGA X. Nodes are supported by 500 bootstraps.

A comparison of 16S rRNA sequences revealed 100% similarity between Bacillus thuringiensis strain UNMSM10RA and Bacillus thuringiensis serovar kurstaki BMB171, and this was corroborated by phylogenetic proximity (Fig. 1). The estimated genome size of B. thuringiensis strain UNMSM10RA was 5,524,132 bp distributed in 449 contigs, with an *N*_50_ value of 61,078 bp and an average G+C content of 35.1%. Genome analysis revealed 5,347 coding sequences, 105 tRNA genes, 15 rRNA genes, 5 noncoding RNA (ncRNA) genes, and 713 pseudogenes. Within protein-coding genes, 31 were detected and identified in the metabolism of heavy metals such as arsenic, cadmium, and copper. Further analyses will be performed and provide more information for understanding the evolution of these Bacillus thuringiensis strains and their potential uses.

### Data availability.

This whole-genome shotgun project has been deposited in NCBI/GenBank under BioProject number PRJNA448338, BioSample number SAMN08828790, GenBank accession number NZ_PZQR00000000, and SRA number SRR10158671. The version described in this paper is the first version, NZ_PZQR01000000.
